# Extracellular vesicles shed by multidrug resistant cells contribute to the identification of SRC inhibitors as chemosensitizers in non-small cell lung cancer

**DOI:** 10.20517/cdr.2025.175

**Published:** 2026-02-10

**Authors:** Bárbara Polónia, Cristina P. R. Xavier, Sara Peixoto da Silva, Chiara Riganti, M. Helena Vasconcelos

**Affiliations:** ^1^i3S - Instituto de Investigação e Inovação em Saúde, Universidade do Porto, Porto 4200-135, Portugal.; ^2^Cancer Drug Resistance Group, IPATIMUP - Institute of Molecular Pathology and Immunology, University of Porto, Porto 4200-135, Portugal.; ^3^Department of Biological Sciences, FFUP - Faculty of Pharmacy of the University of Porto, Porto 4050-313, Portugal.; ^4^Department of Oncology, University of Torino, Torino 10126, Italy.; ^5^Molecular Biotechnology Center “G. Tarone”, University of Torino, Torino 10126, Italy.; ^6^Associate Laboratory i4HB - Institute for Health and Bioeconomy, University Institute of Health Sciences - CESPU, Gandra 4585-116, Portugal.; ^7^UCIBIO - Applied Molecular Biosciences Unit, Toxicologic Pathology Research Laboratory, University Institute of Health Sciences (1H-TOXRUN, IUCS-CESPU), Gandra 4585-116, Portugal.

**Keywords:** Multidrug resistance, non-small cell lung cancer, extracellular vesicles, SRC inhibitors, chemotherapy

## Abstract

**Aim:** Non-small cell lung cancer (NSCLC) represents most lung cancer cases and remains associated with poor outcomes, mainly due to multidrug resistance (MDR). Extracellular vesicles (EVs) are crucial for intercellular communication and significantly influence chemotherapy resistance. This study aimed to characterize the EVs proteome of drug-sensitive and MDR NSCLC cell lines to identify therapeutic targets to counteract MDR.

**Methods:** EVs derived from NSCLC cells were isolated by ultracentrifugation and analyzed for size by nanoparticle tracking analysis, for morphology by transmission electron microscopy, and for EVs markers by Western blotting (WB). Proteomic profiling was performed using liquid chromatography-mass spectrometry (LC-MS), followed by WB validation of relevant proteins. Cell growth and viability were assessed using sulforhodamine B or CellTiter-Glo assays. P-glycoprotein [P-gp, also known as ABCB1: adenosine triphosphate (ATP)-binding cassette subfamily B member 1] activity was determined by rhodamine-123 accumulation assay. SRC-related signaling was investigated by WB.

**Results:** EVs from the multidrug resistant (MDR) derivative of NCI-H460, a human NSCLC cell line, displayed nine up- and eight down-regulated proteins compared with the drug-sensitive parental cells, including reduced SRC. WB results showed higher phosphorylated form of SRC (p-SRC) expression in MDR cells than in sensitive cells. In contrast, EVs from both cell lines had similar expression levels, suggesting selective intracellular retention in MDR cells. The SRC inhibitor bosutinib potentiated the activity of chemotherapeutics that are P-gp substrates in 2D and in 3D spheroids, without affecting the viability of the human lung fibroblast cell line MRC-5. Moreover, bosutinib reduced P-gp activity, likely by downregulating of phosphorylated caveolin-1.

**Conclusion:** These findings show reduced selective packaging of p-SRC into EVs shed by MDR cells (MDR-EVs), suggesting an important role for this protein in the MDR phenotype and its potential as a molecular target. Bosutinib, an SRC inhibitor, might be useful as a chemosensitizer of MDR cells.

## INTRODUCTION

Among all cancer types, lung cancer is the second most frequently diagnosed and the top cause of cancer mortality, responsible for approximately 18% of cancer-related deaths worldwide^[1]^. Lung cancer represents a biologically diverse set of tumors that are conventionally divided into two histologic categories: small cell lung cancer (SCLC) and non-small cell lung cancer (NSCLC). NSCLC accounts for approximately 85% of all cases and has a 5-year survival rate of merely 20%, mainly due to inadequate screening and the delayed onset of symptoms^[2]^. The management of NSCLC typically involves a multidisciplinary approach that includes surgery, radiotherapy, chemotherapy, molecularly targeted therapy, and immunotherapy^[3]^. In fact, conventional chemotherapy, administered as a single drug or as a combined treatment, remains the standard and primary treatment option for NSCLC patients who are not eligible for targeted therapy or immunotherapy. In cases of resectable stage II/III NSCLC, adjuvant chemotherapy based on platinum compounds, typically using cisplatin or carboplatin together with paclitaxel, gemcitabine, or vinorelbine, is routinely used to improve survival outcomes^[3-5]^. However, some cancer patients fail to respond to chemotherapy agents, presenting either intrinsic or acquired chemoresistance^[6]^. Moreover, cross-resistance to a broad array of anticancer drugs with distinct structures and mechanisms of action - known as multidrug resistance (MDR) - poses a particularly challenging obstacle in NSCLC treatment^[7,8]^. The mechanisms behind this MDR phenotype originate either in the host, the tumor microenvironment or the tumor itself^[6]^. Importantly, plasma membrane transporters, such as the adenosine triphosphate (ATP)-binding cassette (ABC) transporter superfamily, are found overexpressed in MDR tumor cells, being important players in drug efflux and strongly associated with MDR^[9]^. The ABC subfamily B member 1 (ABCB1) - commonly known as P-glycoprotein (P-gp) - is one of the most well-characterized members of this family, being a major determinant of drug resistance in cancer^[10]^. Further understanding of the mechanisms that cause cancer cells to resist chemotherapy and identification of novel and more effective therapeutic combinations to counteract the MDR phenotype continues to be a central challenge to lung cancer treatment.

Extracellular vesicles (EVs) are cell-released particles with a size range between 30 and 5,000 nm, enclosed within a lipid bilayer, with no ability to replicate^[11]^. EVs can be naturally found in several biological fluids^[12]^ and were originally considered the “garbage bins” of the cells, being responsible for the removal of unnecessary molecules produced by the cells. However, it is now known that these small particles carry proteins, lipids, DNA, RNA, among other components that have a fundamental role in intercellular communication^[13-16]^ and in a wide range of biological processes^[17]^. Importantly, in various types of cancer, including NSCLC, EVs have been linked to multiple stages of the oncogenic process, from the initiation and progression of tumors to metastasis and resistance to chemotherapy. The content of EVs can either reflect that of their donor cells of origin or result from selective packaging, and may modulate the function of recipient cells^[18-20]^.

This work aimed to analyze the EVs cargo from sensitive and counterpart NSCLC MDR cells (MDR-EVs), identify new molecular targets to overcome MDR and, consequently, repurpose inhibitors of these targets to improve NSCLC treatment.

## METHODS

### Drugs

Doxorubicin (D1515), carboplatin (BP711), paclitaxel (T7402), and vinorelbine (Y0000463) were obtained from Merck Life Science (Darmstadt, Germany). Bosutinib (HY-10158) and ecF506 (HY-112096) were purchased from MedChemExpress (Monmouth Junction, NJ, USA). Paclitaxel, bosutinib, and ecF506 were prepared using dimethyl sulfoxide (DMSO; Merck Life Science, D2650, Darmstadt, Germany) to generate 60 mM stock solutions, while doxorubicin was dissolved to a stock concentration of 2.2 mM. Carboplatin and vinorelbine were dissolved in sterile water for molecular biology (Merck Life Science, 95284, Darmstadt, Germany) at stock concentrations of 26.94 and 10 mM, respectively. Paclitaxel, bosutinib, and ecF506 were stored at -80 °C; vinorelbine and doxorubicin were stored at -20 °C; carboplatin was stored at 4 °C.

### Cell lines

Two NSCLC cell lines - NCI-H460 and A549 - their MDR counterparts - NCI-H460/R and A549-CDR1 - and a non-tumorigenic human lung fibroblast cell line - MRC-5 - were used. NCI-H460, A549, and MRC-5 were purchased from the American Type Culture Collection (ATCC). The NCI-H460/R cell line was kindly provided by Dr. M. Pesic (Belgrade, Serbia)^[21,22]^. The A549-CDR1 cell line was generated by our research group by gradually increasing paclitaxel concentrations in the parental A549 cells. Both NCI-H460/R^[21]^ and A549-CDR1 overexpress the efflux pump P-gp. To preserve chemoresistance, NCI-H460/R cells were exposed monthly to 100 nM doxorubicin, whereas A549-CDR1 cells received weekly treatments with 16.2 nM paclitaxel. NCI-H460 and NCI-H460/R cells were cultured in Roswell Park Memorial Institute (RPMI) 1640 medium supplemented with stable glutamine and 25 mM HEPES (Biowest, Nuaillé, L0496-500). A549, A549-CDR1, and MRC-5 cells were cultured in Dulbecco’s Modified Eagle Medium (DMEM) supplemented with 4.5 g/L glucose and UltraGlutamine™ with sodium pyruvate (Lonza, BE12-604F). All media contained 10% fetal bovine serum (FBS; Biowest, Nuaillé, S181H-500), except for the sulforhodamine B (SRB) assay, where 5% FBS was used. All cell lines were maintained at 37 °C in a humidified incubator with 5% CO_2_. Experiments were performed with cells in exponential growth and over 90% viability. Cell lines were genotyped and frequently monitored for mycoplasma contamination (Cell Culture and Genotyping Service, i3S).

### Extracellular vesicles isolation

NCI-H460 and NCI-H460/R cell lines were cultured in RPMI-1640 medium supplemented with 10% EVs-depleted FBS (previously ultracentrifuged at 100,000 *g*, 4 °C, for at least 16 h) and 1% antibiotic/antimycotic solution (Merck Life Science, A5955) for 72 h. The conditioned medium was then collected and processed by differential centrifugation as previously described by Théry *et al.*^[23]^. Briefly, the supernatant was centrifuged at 4 °C as follows: (i) 10 min at 2,000 *g*; (ii) 30 min at 10,000 *g* (high-speed centrifuge, Beckman Coulter); (iii) 1 h 15 min at 100,000 *g* (ultracentrifuge, Beckman Coulter). The pellet was washed in phosphate-buffered saline (PBS) and ultracentrifuged again for 1 h 15 min at 100,000 *g*. The final pellet was resuspended in PBS. EVs were characterized in terms of size (by nanoparticle tracking analysis, NTA), morphology (by transmission electron microscopy, TEM), and the presence of classical EVs markers (by Western blotting, WB).

### NTA

EVs were diluted in PBS (1:1,000) to achieve an optimal concentration of 10^7^-10^9^ particles/mL and loaded into a NanoSight NS300 (Malvern Instruments Ltd., Malvern, UK). Three 30 s videos per sample were recorded with the following specifications: camera type, sCMOS; laser type, Blue488; camera level, 15-16; slider shutter, 1,206-1,300; slider gain, 366-512; frame rate (FPS), 25.0; temperature, 21.9-25.9 °C; viscosity (water), 0.870-0.955 cP; syringe pump speed, 40. Videos were analyzed using NanoSight NTA Software (version 3.2, Dev Build 3.2.16) with the following settings: detection threshold, 5; blur size, auto; max jump distance, auto (12.7-31.7 pixels). The mean vesicle size (nm) was determined.

### TEM

EVs were diluted 1:2 in a solution of 20 mM HEPES (Merck Life Science, H-4034) and 4% (w/v) sucrose (Merck Life Science, S0389-500G). The preparations were transferred onto Formvar–carbon–coated electron microscopy grids and allowed to stand at room temperature (RT) in the dark for 2 min. The grids were then stained with 5% uranyl acetate and examined using a JEM-1440 transmission electron microscope (Jeol) operated at 80 kV. Digital images were captured with an Orius 1100 W camera (Gatan). Representative TEM images were acquired from at least one independent experiment for each sample. Data acquisition was performed by the Histology and Electron Microscopy Service, i3S, Porto, Portugal.

### Proteomic analysis

The protein content of EVs (30 μg) derived from NCI-H460 and NCI-H460/R cell lines was analyzed in three independent experiments by liquid chromatography with tandem mass spectrometry (LC-MS/MS). Samples were processed using a previously described solid-phase-enhanced sample-preparation (SP3) procedure, followed by enzymatic digestion with Trypsin/LysC^[24]^. Nano-LC-MS/MS was performed to identify and quantify proteins according to a previously published protocol^[25,26]^. Reviewed proteomes of *Homo sapiens* (2021_03, 20,371 entries) and *Bos taurus* (2021_03, 6,014 entries) from the UniProt database were used for protein identification, along with a human spectral library (NIST_Human_Orbitrap_HCD_20160923). Data analysis was carried out using Proteome Discoverer 2.5.0.400 software (Thermo Scientific). Label-free protein quantification was performed using the Minora feature detector node with the following parameters: (i) peptides - unique plus razor; (ii) precursor abundance based on intensity; (iii) normalization mode based on total peptide amount; (iv) pairwise protein ratio calculation; (v) hypothesis testing using a *t*-test (background-based). Additional parameters are described in Supplementary Text 1. All LC-MS/MS procedures and raw data processing were performed by the Proteomics Scientific Platform, i3S, Porto, Portugal. Identified proteins were compared with entries in two open EVs databases: Vesiclepedia and ExoCarta.

### Bioinformatics analysis using The Cancer Genome Atlas

To assess the impact of SRC expression on overall survival (OS), NSCLC cohort data were downloaded from The Cancer Genome Atlas (TCGA; https://cancergenome.nih.gov/), comprising 994 patients with gene expression data quantified as FPKM (fragments per kilobase of transcript per million mapped reads). Survival information was extracted from the corresponding clinical files. Survival analyses were performed in R using the “survival” and “survminer” packages. Expression values were categorized as “high” or “low” based on the best separation cutoff method, as employed by the Human Protein Atlas. This cutoff corresponds to the FPKM value within the second quartile that produces the lowest log-rank *P*-value when comparing survival between the two groups.

### Protein extraction and quantification

Cell pellets were collected, washed with PBS, and centrifuged at 240 *g* for 5 min at 4 °C. Cells were then lysed using Wyman’s Buffer (1% NP-40, 0.1 M Tris-HCl, pH 8.0, 0.15 M NaCl, and 5 mM EDTA) supplemented with protease and phosphatase inhibitor cocktails for 30 min at 4 °C. Protein lysates were obtained after centrifugation at 13,200 *g* for 10 min at 4 °C. Protein quantification of cells and EVs was performed using a modified Lowry protocol (DC™ Protein Assay kit; Bio-Rad, Hercules, 5000116), with BSA as the protein standard. Absorbance was measured using a microplate reader (Synergy PowerWave XS, BioTek Instruments Inc.) with excitation at 488 nm and emission detection at 655 nm.

### WB

A total of 20 µg of protein from both cell and EVs samples was denatured in loading buffer [1 M Tris-HCl, pH 6.8; 10% sodium dodecyl sulfate (SDS); 85% glycerol; β-mercaptoethanol; 1% bromophenol blue] and heated at 95 °C for 5 min. Samples were resolved by size using Sodium Dodecyl Sulfate–Polyacrylamide Gel Electrophoresis (SDS-PAGE) (Mini-PROTEAN® Tetra Vertical Electrophoresis Cell, Bio-Rad) and subsequently transferred onto a nitrocellulose membrane with the Mini Trans-Blot® cell system (Bio-Rad). Membranes were stained with Ponceau S solution. Blocking was performed for at least 30 min in one of the following: 5% (w/v) non-fat dry milk in TBS-T (Tris-buffered saline, pH 7.4, with 0.1% Tween-20), 5% (w/v) bovine serum albumin (BSA) in TBS-T, or EveryBlot Blocking Buffer (Bio-Rad). Membranes were then incubated with the following primary antibodies for 90 min at RT or overnight at 4 °C: mouse anti-Alix (sc-53540), mouse anti-syntenin-1 (sc-100336), mouse anti-p-SRC (sc-81521), mouse anti-SRC (sc-8056), mouse anti-MDR1 (sc-55510), mouse anti-connexin 43 (sc-271837), mouse anti-β-actin (sc-47778), rabbit anti-caveolin-1 (sc-894), mouse anti-p-caveolin-1 (sc-373836) from Santa Cruz Biotechnology; rabbit anti-CD9 (EXOAB-CD9A-1) from System Biosciences; and rabbit anti-alpha actinin 4 (GTX113116) from GeneTex. Membranes were washed in TBS-T, followed by incubation with secondary antibodies for 1 h at RT: anti-mouse (NA931V) or anti-rabbit (NA934V) from GE Healthcare. Signals were detected using enhanced chemiluminescence (ECL) WB Detection Reagent, Amersham Hyperfilm ECL, or Clarity Max Western ECL Substrate (Bio-Rad) and visualized with a Fuji Medical Film Processor or ChemiDoc™ Touch Imaging System (Bio-Rad). Band quantification was performed using Image Lab™ Software version 6.0.1 (Bio-Rad, Hercules, CA, USA).

### SRB assay

The SRB assay was used to determine the concentration that inhibits 50% of cell growth (GI_50_ concentration) and to assess drug cytotoxicity on the MRC-5 cell line, following a previously described protocol^[27,28]^. Cells were plated in 96-well plates at an optimal density of 5 × 10^4^ cells/mL for 24 h. Cells were then treated with the desired drug concentrations and corresponding controls for an additional 48 h. Two plates were used: one analyzed at the time of treatment (T0) and another at 48 h post-treatment (T48 h). For long-term cytotoxicity assessment on MRC-5 cells, a third timepoint (T+172 h) was included: after 48 h, the treatment-containing medium was removed and replaced with fresh medium, and cell growth was measured.

After the incubation periods, cells were fixed with 10% (w/v) trichloroacetic acid for at least 1 h at 4 °C and washed with distilled water. Cells were then stained with 0.4% (w/v) SRB in 1% (v/v) acetic acid for 30 min. Unbound dye was removed by washing with 1% (v/v) acetic acid, and the plates were allowed to dry at RT. Bound SRB was solubilized with 10 mM Tris base, and absorbance was measured at 510 nm using a multiplate reader (Synergy PowerWave XS, BioTek Instruments Inc.) with Gen5™ software.

### Trypan blue exclusion assay

Cell viability was assessed using the trypan blue exclusion method. Following drug treatment, equal volumes of cell suspension and 0.2% (v/v) trypan blue solution (Merck Life Science, T8154) were mixed. Viable cells remained unstained, whereas non-viable cells appeared blue. Cell counts were performed manually using a Neubauer hemocytometer.

### Rhodamine-123 accumulation assay

Cells were plated at a concentration of 1 × 10^5^ cells/mL in a 6-well plate for 24 h. The desired drug concentrations and the vehicle control (DMSO, at the highest concentration used) were then added. Verapamil (20 µM) was included as a positive control. All conditions were performed in duplicate, in the presence or absence of 1 µM rhodamine-123 (Merck Life Science, R8004). After 24 h of incubation, cells were centrifuged at 240 *g* for 5 min, resuspended in 300 µL of PBS, and kept in the dark until flow cytometry analysis using a BD Accuri™ C6 Flow Cytometer (BD Biosciences). A minimum of 20,000 events were recorded per condition.

### Combination index determination

To determine whether the combination of drugs exhibited a synergistic, additive, or antagonistic effect, the combination index (CI) was calculated as^[29]^:

**Figure:**
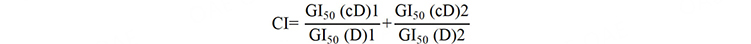


where GI_50_ (cD)1 and GI_50_ (cD)2 represent the GI_50_ concentrations of drug 1 and drug 2, respectively, when used in combination, and GI_50_ (D)1 and GI_50_ (D)2 refer to the GI_50_ concentrations of each drug individually. A CI less than 1 indicates a synergistic effect, a CI equal to 1 indicates an additive effect, and a CI greater than 1 indicates an antagonistic effect.

### 3D spheroids of NSCLC cells under drug treatment

The 3D spheroids were generated using 3D Petri Dish (Merck Life Science, MICROTISSUES technology, MicroTissues Inc.), as previously described^[30]^. Agarose micromolds were prepared with 2% agarose in 0.9% NaCl and equilibrated in RPMI-1640 medium supplemented with 10% FBS for at least 24 h in a 12-well plate. Cells were seeded into the micromolds at a concentration of 750 cells per spheroid and allowed to settle for 30 min before adding the final medium volume. Spheroid formation was monitored every 24 h using an inverted light microscope (Leica DMi1, Leica Biosystems). After 72 h, the desired drug concentrations and vehicle control (DMSO at the highest concentration used) were added. Cell viability was assessed at 72 and 96 h using the CellTiter-Glo® 3D Cell Viability Assay (Promega), according to the manufacturer’s instructions. Luminescence was measured with a multiplate reader (Synergy™ Mx, Biotek Instruments Inc.). Spheroid morphology was monitored every 24 h using an inverted light microscope. The area of at least six spheroids per timepoint and condition was measured using AnaSP software^[31]^, and the area increase relative to the timepoint at which the drugs were added (T0) was calculated.

### Statistical analysis

All data were obtained from at least three independent experiments, and results are expressed as mean ± standard error of the mean (SEM). Statistical analysis was performed using GraphPad Prism 8.0, with a two-tailed unpaired *t*-test. For proteomic analysis, *P*-values comparing the two groups were calculated using Student’s *t*-test and subsequently adjusted for multiple testing across all quantified proteins using the Benjamini–Hochberg false discovery rate (FDR) procedure. Statistical significance was defined as *P* < 0.05.

## RESULTS

### EVs were successfully isolated from sensitive NCI-H460 and counterpart MDR NCI-H460/R cell lines

**Figure 1:**
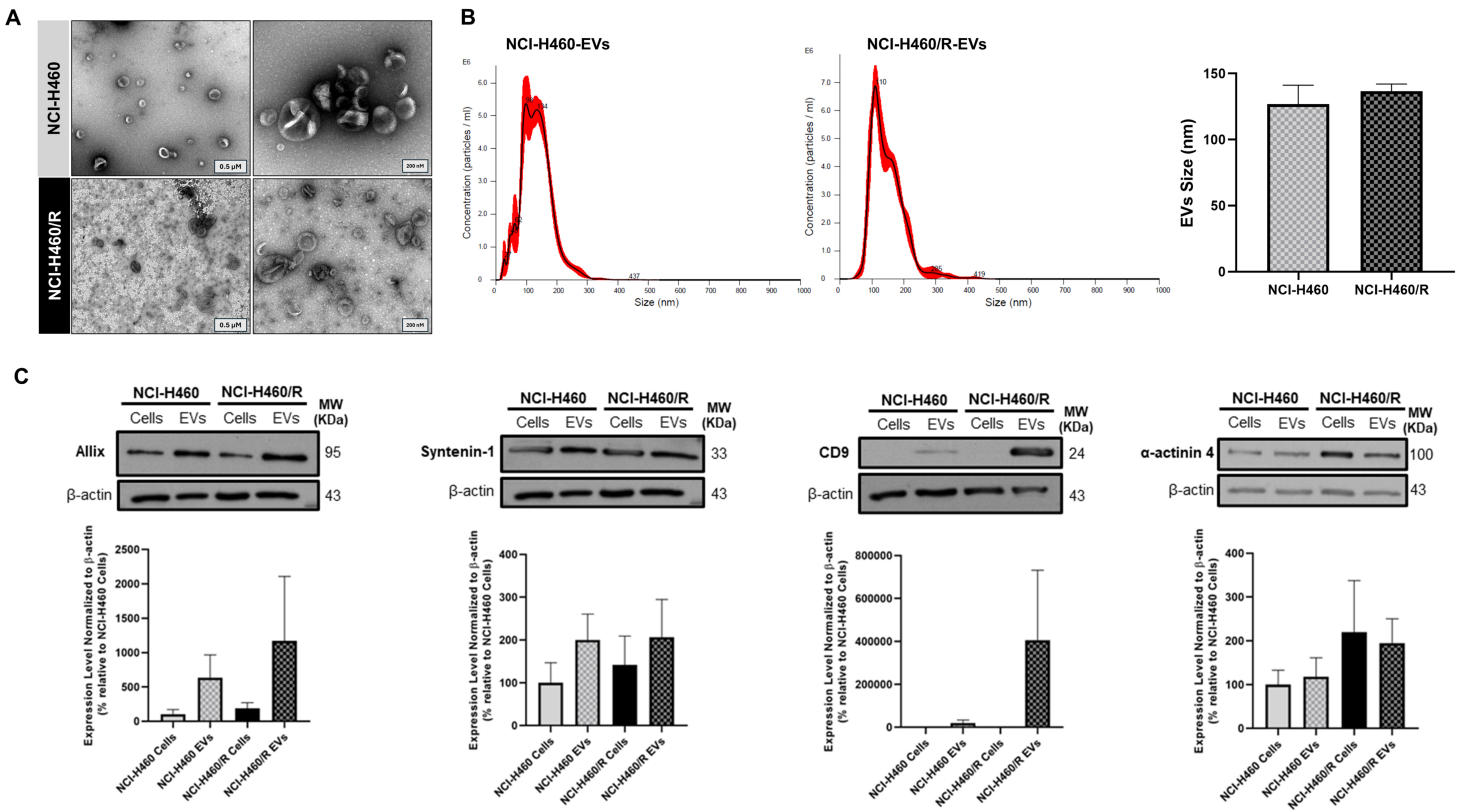
Characterization of EVs isolated by differential ultracentrifugation from the NSCLC cell line NCI-H460 and its multidrug resistant counterpart NCI-H460/R. (A) Representative TEM images of EVs (left: scale bar = 0.5 µM; right: scale bar = 200 nm). Results are representative of one experiment; (B) Particle size distribution and mean size of EVs, analyzed by NTA. Data are represented as mean ± SEM of at least three independent experiments; (C) EV markers detected by WB in EVs and respective cells. Data are represented as mean ± SEM of at least three independent experiments. EVs: Extracellular vesicles; NSCLC: non-small cell lung cancer; TEM: transmission electron microscopy; NTA: nanoparticle tracking analysis; SEM: standard error of the mean; WB: Western blotting.

The EVs isolated from the sensitive NCI-H460 and the MDR counterpart NCI-H460/R NSCLC cell lines displayed the size and morphology expected for EVs. TEM images showed an EV-typical cup-shaped structure in particles isolated from both cell lines [Figure 1A]. NTA results demonstrated that EVs from both cell lines ranged in size from 50 to 300 nm, with a mean size of approximately 130 nm, which is within the expected range for EVs [Figure 1B]. As recommended by the MISEV guidelines^[11,32]^, protein-content characterization of the isolated EVs was performed by WB [Figure 1C]. The cytosolic proteins Alix and Syntenin-1, as well as the transmembrane protein CD9, were enriched in the EVs compared to the respective cells. α-actinin 4 levels did not differ between cells and EVs. These results indicate that EVs were successfully isolated, presenting the expected size, morphology, and EVs markers.

### EVs shed by sensitive and MDR counterpart cell lines presented differentially expressed proteins

**Figure 2:**
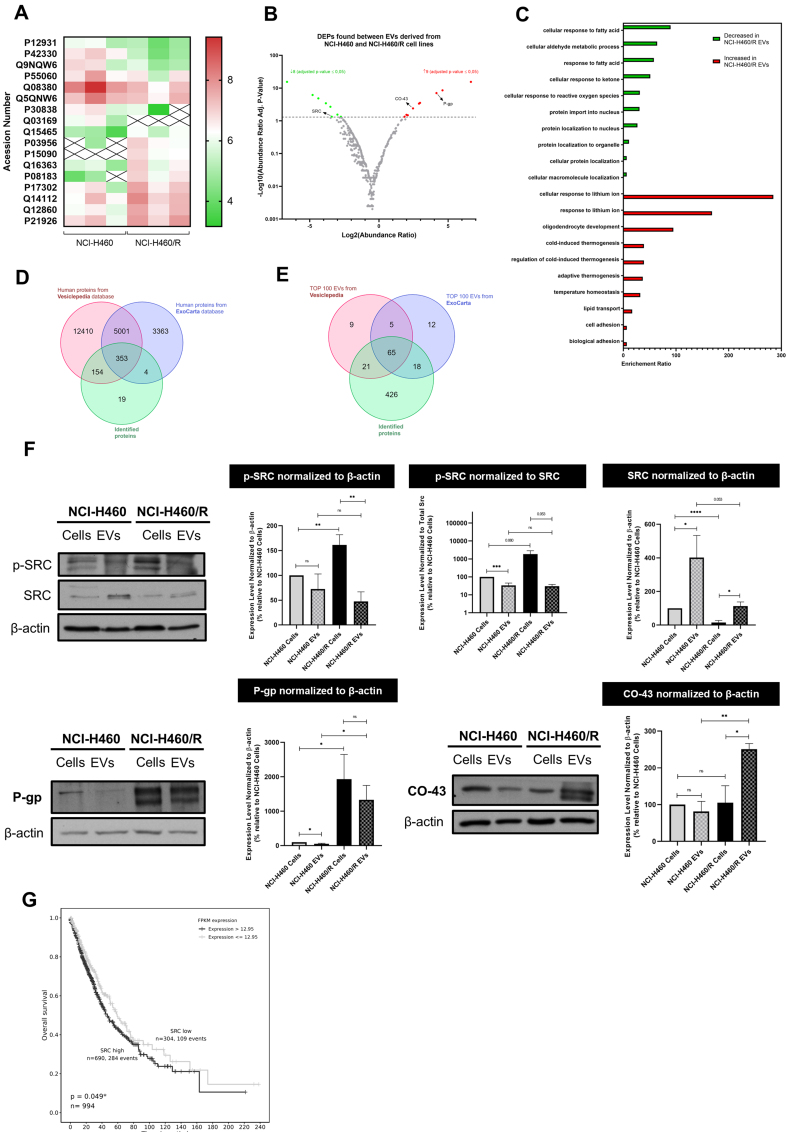
Proteomic analysis of EVs isolated from NCI-H460 and NCI-H460/R cell lines and validation of the results. (A) Heat map of DEPs between EVs isolated from the sensitive and multidrug resistant cell lines; (B) Volcano plot of all identified proteins in EVs isolated from both cell lines; (C) Gene ontology analysis showing the biological processes associated with proteins present in EVs isolated from both cell lines; (D) Venn diagram comparing proteins identified in this study with those in the Vesiclepedia and ExoCarta databases; (E) Comparative analysis between the Top 100 EV proteins identified in this study and those reported in Vesiclepedia and ExoCarta databases; (F) Analysis of the levels of proteins found in cells and EVs from counterpart sensitive and MDR cells, determined by WB. Representative blots and quantitative analysis (mean ± SEM) are from at least three independent experiments. Statistical significance was assessed using an unpaired Student’s *t*-test: ^*^P < 0.05; ^**^P < 0.01; ^***^P < 0.001; ^****^P < 0.0001; ns: non-significant; (G) Kaplan-Meier curves showing OS according to SRC gene expression in NSCLC patients. EVs: Extracellular vesicles; DEPs: differentially expressed proteins; MDR: multidrug resistance; WB: Western blotting; SEM: standard error of the mean; OS: overall survival; NSCLC: non-small cell lung cancer; p-SRC: phosphorylated form of SRC; P-gp: P-glycoprotein.

A proteomic analysis of EVs derived from the chemosensitive NCI-H460 cell line and its resistant counterpart, NCI-H460/R, was performed using LC-MS. Results showed that, out of a total of 530 identified proteins, 17 were differentially expressed (DEPs) between EVs isolated from the sensitive and MDR cell lines. Among these proteins, 8 were significantly decreased and 9 were increased in EVs derived from the MDR cell line compared to EVs from the sensitive parental line, as shown in the heat map [Figure 2A] and volcano plot [Figure 2B]. Table 1 lists all the DEPs.

**Table 1:** DEPs in EVs derived from NCI-H460 and NCI-H460/R cell lines

	**UniProt accession number**	**Protein name**	**Gene symbol**	**Unique peptides**	**Mean abundance (normalized)**			**Abundance ratio**	
					**NCI-H460**	**NCI-H460/R**	**Fold change resistant/sensitive**	**Resistant/sensitive**	**Adjusted *P*-value using Benjamini-Hochberg correction**
**Increased in NCI-H460/R derived EVs**	P15090	Fatty acid-binding protein, adipocyte	FABP4	3	Not found	3.69 × 10^6^	N/A	100	3.50 × 10^-16^
	P08183	P-glycoprotein	ABCB1	7	8.12 × 10^4^	5.24 × 10^6^	64.53	17.85	9.60 × 10^-8^
	P03956	Interstitial collagenase	MMP1	5	6.35 × 10^4^	4.77 × 10^6^	75.11	24.14	2.99 × 10^-9^
	Q15465	Sonic hedgehog protein	SHH	3	6.82 × 10^4^	1.11 × 10^6^	16.33	4.24	3.32 × 10^-2^
	Q12860	Contactin-1	CNTN1		1.84 × 10^6^	2.47 × 10^7^	13.48	7.83	2.92 × 10^-4^
	Q14112	Nidogen-2	NID2	32	8.86 × 10^6^	5.07 × 10^7^	5.72	3.60	4.63 × 10^-2^
	P21926	CD9 antigen	CD9	5	8.77 × 10^6^	4.45 × 10^7^	5.07	7.42	4.41 × 10^-4^
	Q16363	Laminin subunit alpha-4	LAMA4	11	4.23 × 10^5^	5.15 × 10^6^	12.19	3.97	2.99 × 10^-2^
	P17302	Gap junction alpha-1 protein	GJA1	10	2.79 × 10^6^	2.16 × 10^7^	7.75	5.50	4.00 × 10^-3^
**Decreased in NCI-H460/R derived EVs**	P30838	Aldehyde dehydrogenase family 3 member A1	ALDH3A1	2	3.03 × 10^6^	4.42 × 10^5^	0.15	0.13	2.82 × 10^-2^
	P42330	Aldo-keto reductase family 1 member C3	AKR1C3	5	6.65 × 10^6^	1.07 × 10^5^	0.02	0.05	1.44 × 10^-5^
	Q9NQW6	Anillin	ANLN	7	4.89 × 10^6^	3.08 × 10^5^	0.06	0.07	3.72 × 10^-4^
	P55060	Exportin-2	CSE1L	2	2.30 × 10^7^	9.85 × 10^6^	0.43	0.09	2.09 × 10^-3^
	Q08380	Galectin-3-binding protein	LGALS3BP	19	9.57 × 10^8^	1.46 × 10^7^	0.02	0.04	7.28 × 10^-7^
	Q5QNW6	Histone H2B type 2-F	H2BC18	4	8.11 × 10^7^	1.61 × 10^7^	0.20	0.15	4.63 × 10^-2^
	P12931	Proto-oncogene tyrosine-protein kinase SRC	SRC	2	8.56 × 10^5^	2.40 × 10^5^	0.28	0.09	4.63 × 10^-2^
	Q03169	Tumor necrosis factor alpha-induced protein 2	TNFAIP2	2	1.44 × 10^6^	Not found	N/A	0.01	3.50 × 10^-16^

At least three independent experiments were performed to calculate the mean abundance, the respective abundance ratio and adjusted *P*-values. DEPs: Differentially expressed proteins; EVs: extracellular vesicles; N/A: not applicable.

Gene ontology analysis [Figure 2C] revealed that the most relevant biological processes associated with the decreased proteins in MDR-EVs include cellular response to fatty acids and reactive oxygen species, cellular aldehyde metabolic processes, and protein localization to the nucleus. In contrast, biological processes enriched in MDR-EVs include lipid transport, cell adhesion, biological adhesion, and regulation of cold-induced thermogenesis.

The identified proteins in this study were compared with the public databases Vesiclepedia and ExoCarta. Of the 530 identified proteins, 511 are present in the databases - 154 in Vesiclepedia and 4 in ExoCarta - while 19 proteins were uniquely identified in our study [Figure 2D]. Additionally, a total of 10 identified proteins are included in the top 100 EV proteins from at least one of the databases [Figure 2E].

Further validation of the proteomics analysis was performed by WB [Figure 2F], along with a comparison of protein expression levels between EVs and their cells of origin. Consistent with their resistance phenotype, MDR cells and their EVs displayed increased levels of the efflux pump P-gp compared with the sensitive counterparts. Interestingly, MDR cells showed reduced total SRC levels compared with their sensitive parental line, whereas the activated form, phospho-SRC (p-SRC), was higher in MDR cells than in sensitive cells. Thus, MDR cells contained less SRC but more active p-SRC. In EVs, however, this pattern was not maintained. Although SRC was also reduced in MDR-derived EVs, p-SRC levels were comparable between EVs from MDR and sensitive cells. These results suggest that p-SRC may be retained by MDR cells and could be relevant for the MDR phenotype. Additionally, the protein CO-43 - known to be associated with p-SRC inhibition^[33,34]^ - is enriched in MDR-EVs compared with MDR cells, whereas similar levels of CO-43 are found in sensitive cells and their EVs, suggesting selective packaging of CO-43 into MDR-EVs.

Altogether, these data suggest that p-SRC may be a promising molecular target to overcome MDR and that SRC inhibitors may sensitize MDR cells to chemotherapy. Importantly, Kaplan–Meier survival analysis [Figure 2G], performed using the TCGA database, revealed that NSCLC patients with high SRC expression (> 12.95 FPKM) exhibited worse OS compared with those with low SRC expression (≤ 12.95 FPKM). The log-rank test showed a statistically significant difference between the survival curves (*P* = 0.049). These findings indicate that higher SRC expression correlates with worse prognosis in this cohort.

### SRC inhibitors presented a synergistic effect with conventional chemotherapeutics in MDR NCI-H460/R cells

Two SRC pathway inhibitors, bosutinib and ecF506, were selected to evaluate their chemosensitizing effects in MDR cells. Bosutinib inhibited the growth of NCI-H460 and NCI-H460/R cell lines, with GI_50_ concentrations of 1.43 and 1.68 µM, respectively. ecF506 showed GI_50_ concentrations of 30.55 and 23.47 µM for NCI-H460 and NCI-H460/R cells, respectively [Table 2].

**Table 2:** GI_50_ concentrations (µM) of bosutinib and ecF506 in NCI-H460, NCI-H460/R, A549, and A549-CDR1 cell lines

**GI_50_ concentration (µM)**					
		**Cell lines**			
		**NCI-H460**	**NCI-H460/R**	**A549**	**A549-CDR1**
**Drugs**	Bosutinib	1.43 ± 0.06	1.68 ± 0.08	1.12 ± 0.87	2.89 ± 0.22
	ecF506	30.55 ± 2.36	23.47 ± 0.94	7.75 ± 0.51	10.28 ± 0.31

Results are expressed as mean ± SEM from at least three independent experiments. SEM: Standard error of the mean.

**Figure 3:**
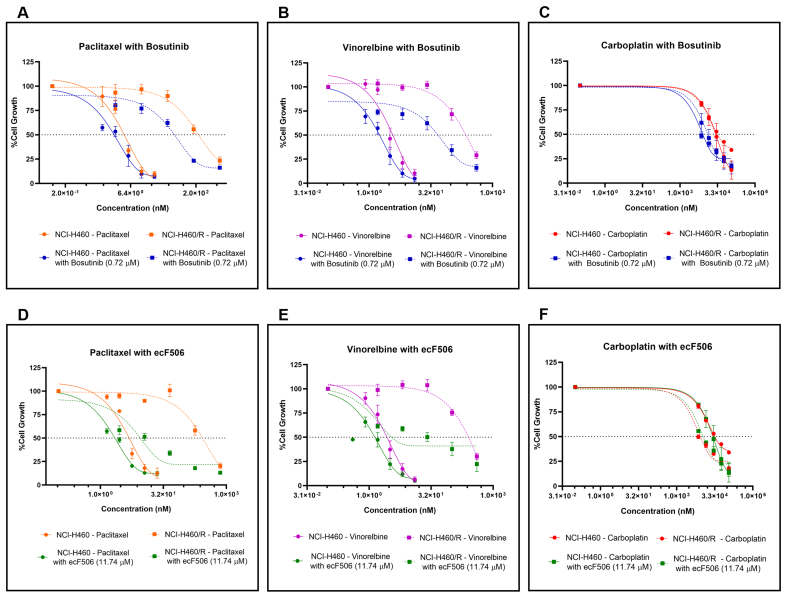
Effect of conventional chemotherapeutic drugs used in NSCLC treatment (paclitaxel, vinorelbine, or carboplatin), alone or in combination with bosutinib or ecF506, on the percentage of cell growth in sensitive NCI-H460 and MDR NCI-H460/R counterpart cell lines, assessed by the SRB assay. Serial dilutions of the chemotherapeutic drugs were applied with or without 0.72 µM bosutinib or 11.74 µM ecF506. The vehicle at the highest tested concentration was used as control. Combinations tested: (A) paclitaxel + bosutinib; (B) vinorelbine + bosutinib; (C) carboplatin + bosutinib; (D) paclitaxel + ecF506; (E) vinorelbine + ecF506; (F) carboplatin + ecF506. Results represent mean ± SEM of at least three independent experiments. NSCLC: Non-small cell lung cancer; MDR: multidrug resistance; SRB: sulforhodamine B; SEM: standard error of the mean.

Combination studies were then performed in both sensitive NCI-H460 and MDR NCI-H460/R cells. For each cell line, bosutinib or ecF506 was combined with five serial dilutions of conventional chemotherapeutic drugs used in NSCLC treatment - paclitaxel and vinorelbine (P-gp substrates) or carboplatin (non-P-gp substrate). Fixed concentrations of bosutinib (0.72 μM) and ecF506 (11.74 μM), corresponding to half of the lowest GI_50_ concentrations previously determined, were used [Figure 3]. In drug-sensitive NCI-H460 cells, the GI_50_ of paclitaxel or vinorelbine combined with bosutinib [Figure 3A and B] was lower than that of each drug alone, although the differences did not reach statistical significance. In contrast, in MDR NCI-H460/R cells, both combinations caused a statistically significant reduction in GI_50_ compared with single-drug treatments. Furthermore, CI analysis indicated synergism (CI < 1) in MDR cells but not in sensitive cells (CI > 1) for both drug combinations [Table 3].

**Table 3:** GI_50_ concentrations (nM) of paclitaxel, vinorelbine, or carboplatin, alone or in combination with 0.72 µM bosutinib or 11.74 µM ecF506, in NCI-H460 and NCI-H460/R cells

	**NCI-H460**		***P*-value**	**NCI-H460/R**		***P*-value**
**GI_50_ Concentration (nM)**		**CI**			**CI**	
Paclitaxel	4.03 ± 0.39			234.74 ± 41.21		
Paclitaxel with Bosutinib (0.72 µM)	3.49 ± 1.08	1.31	ns	69.50 ± 6.27	0.68	**
Paclitaxel	4.48 ± 0.23			242.27 ± 36.68		
Paclitaxel with ecF506 (11.74 µM)	2.21 ± 0.37	0.91	**	11.05 ± 3.48	0.66	***
Vinorelbine	3.52 ± 0.49			199.34 ± 26.86		
Vinorelbine with Bosutinib (0.72 µM)	2.23 ± 0.48	1.03	ns	50.88 ± 11.48	0.66	***
Vinorelbine	3.12 ± 0.51			213.40 ± 21.90		
Vinorelbine with ecF506 (11.74 µM)	1.89 ± 0.28	0.98	ns	22.14 ± 6.63	0.67	***
Carboplatin	3.65 × 10^4^ ± 3.91 × 10^3^			2.77 × 10^4^ ± 5.31 × 10^3^		
Carboplatin with Bosutinib (0.72 µM)	8.13 × 10^3^ ± 4.26 × 10^2^	0.65	**	1.41 × 10^4^ ± 2.47 × 10^3^	0.89	ns
Carboplatin	3.65 × 10^4^ ± 3.91 × 10^3^			2.74 × 10^4^ ± 5.02 × 10^3^		
Carboplatin with ecF506 (11.74 µM)	7.07 × 10^3^ ± 1.31 × 10^3^	0.67	**	1.19 × 10^4^ ± 9.91 × 10^2^	0.93	*

CI and statistical significance of combinations *vs.* single-drug treatments were determined using an unpaired Student’s *t*-test. ^*^P < 0.05; ^**^P < 0.01; ^***^P < 0.001; ns: non-significant. Results represent mean ± SEM of at least three independent experiments. CI: Combination index; SEM: standard error of the mean.

Conversely, in both sensitive and MDR cell lines, the combination of carboplatin with bosutinib [Figure 3C] resulted in a lower GI_50_ concentration compared with carboplatin alone, with statistical significance observed in the sensitive cells. CI analysis indicated synergy (CI < 1) in both cell lines, although the synergistic effect was markedly stronger in sensitive cells than in resistant cells - an inverse pattern compared to that observed with paclitaxel or vinorelbine combined with bosutinib [Table 3]. Furthermore, the combinations of paclitaxel, vinorelbine, or carboplatin with ecF506 [Figure 3D-F] also yielded lower GI_50_ concentrations than each drug alone, in both sensitive and MDR cells. CI analysis confirmed synergism (CI < 1) for all combinations with ecF506, with the strongest synergy observed in MDR cells for paclitaxel or vinorelbine combined with ecF506. Interestingly, the combination of carboplatin with ecF506 showed its greatest synergistic effect in sensitive cells [Table 3]. These findings may be explained by carboplatin not being a substrate of the drug efflux pump P-gp, unlike paclitaxel and vinorelbine. A detailed statistical analysis for the effect of each concentration for all drug combinations, compared with individual drugs and the vehicle, is presented in Supplementary Figure 1.

### Bosutinib did not increase the cytotoxic effect of paclitaxel, vinorelbine and carboplatin in a human non-tumorigenic cell line

**Figure 4:**
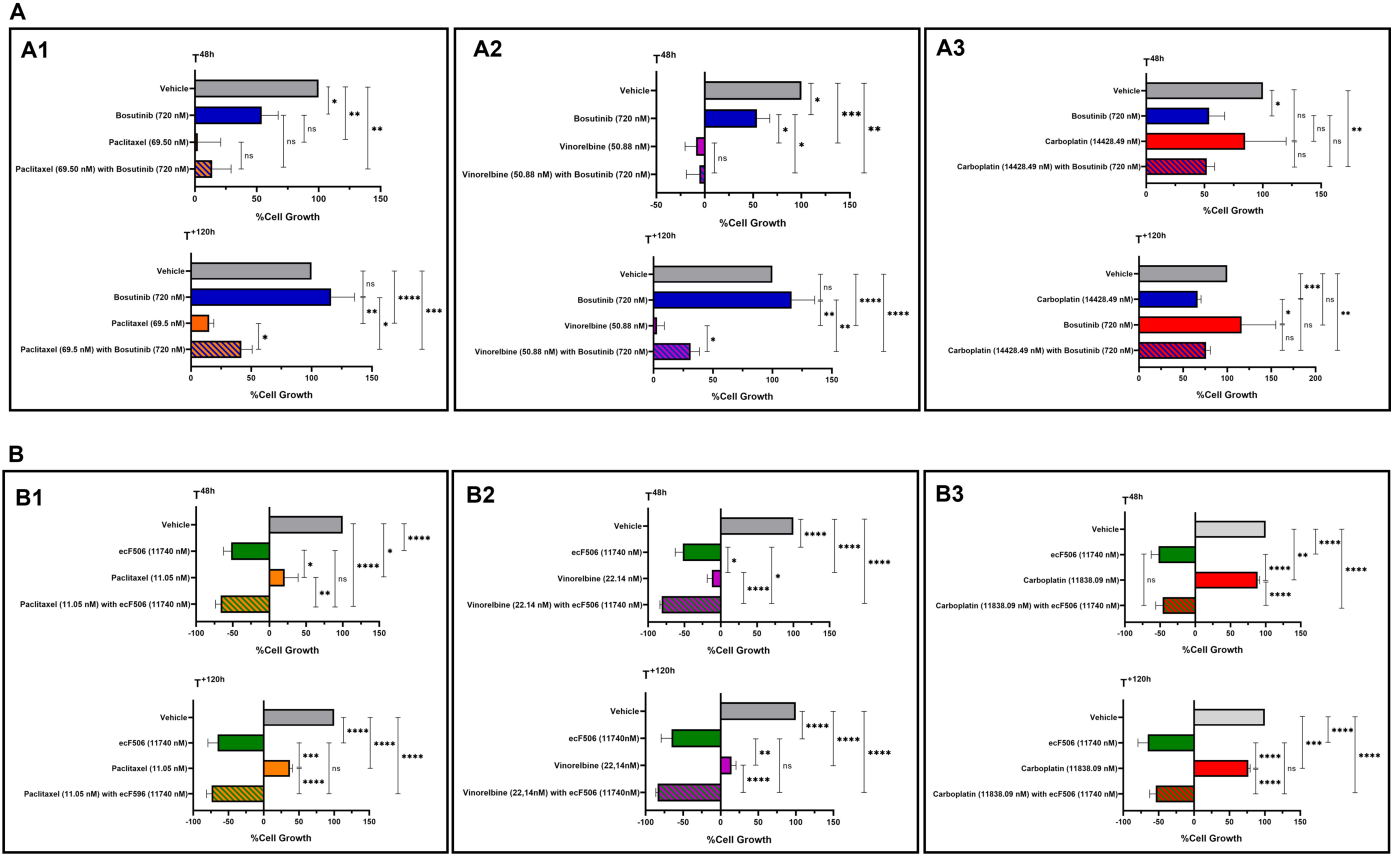
Cytotoxic effects of paclitaxel (A1 and B1), vinorelbine (A2 and B2) or carboplatin (A3 and B3) alone or in combination with bosutinib (A) or ecF506 (B), in the non-tumorigenic MRC-5 cell line. Cell growth was assessed after 48 h of treatment and after an additional 120 h in drug-free medium. The effect of the vehicle at the highest concentration tested was also evaluated. Results are expressed as the percentage of cell growth and represent the mean ± SEM of at least three independent experiments. Statistical significance was determined using an unpaired Student’s *t*-test: ^*^P < 0.05; ^**^P < 0.01; ^***^P < 0.001; ^****^P < 0.0001; ns: non-significant. SEM: Standard error of the mean.

The cytotoxic effect of bosutinib or ecF506, alone and in combination with conventional chemotherapeutic drugs, was assessed in the non-tumorigenic cell line MRC-5 using the SRB assay [Figure 4]. For bosutinib, results showed no changes in the growth inhibition of MRC-5 cells following treatment with drug combinations containing bosutinib [Figure 4A1-3] compared with chemotherapy alone, both at 48 h post-treatment and after removing the drugs for an additional 120 h. This indicates that bosutinib combinations do not increase the cytotoxicity of conventional chemotherapeutic agents. In contrast, combinations containing ecF506 [Figure 4B1-3] showed a significant cytotoxic effect, as ecF506 alone or in combination significantly decreased MRC-5 cell growth compared with conventional chemotherapy alone at both time points tested. Consequently, ecF506 was excluded from further experiments.

### The synergistic effect of bosutinib was confirmed in another pair of sensitive and MDR NSCLC cells

**Figure 5:**
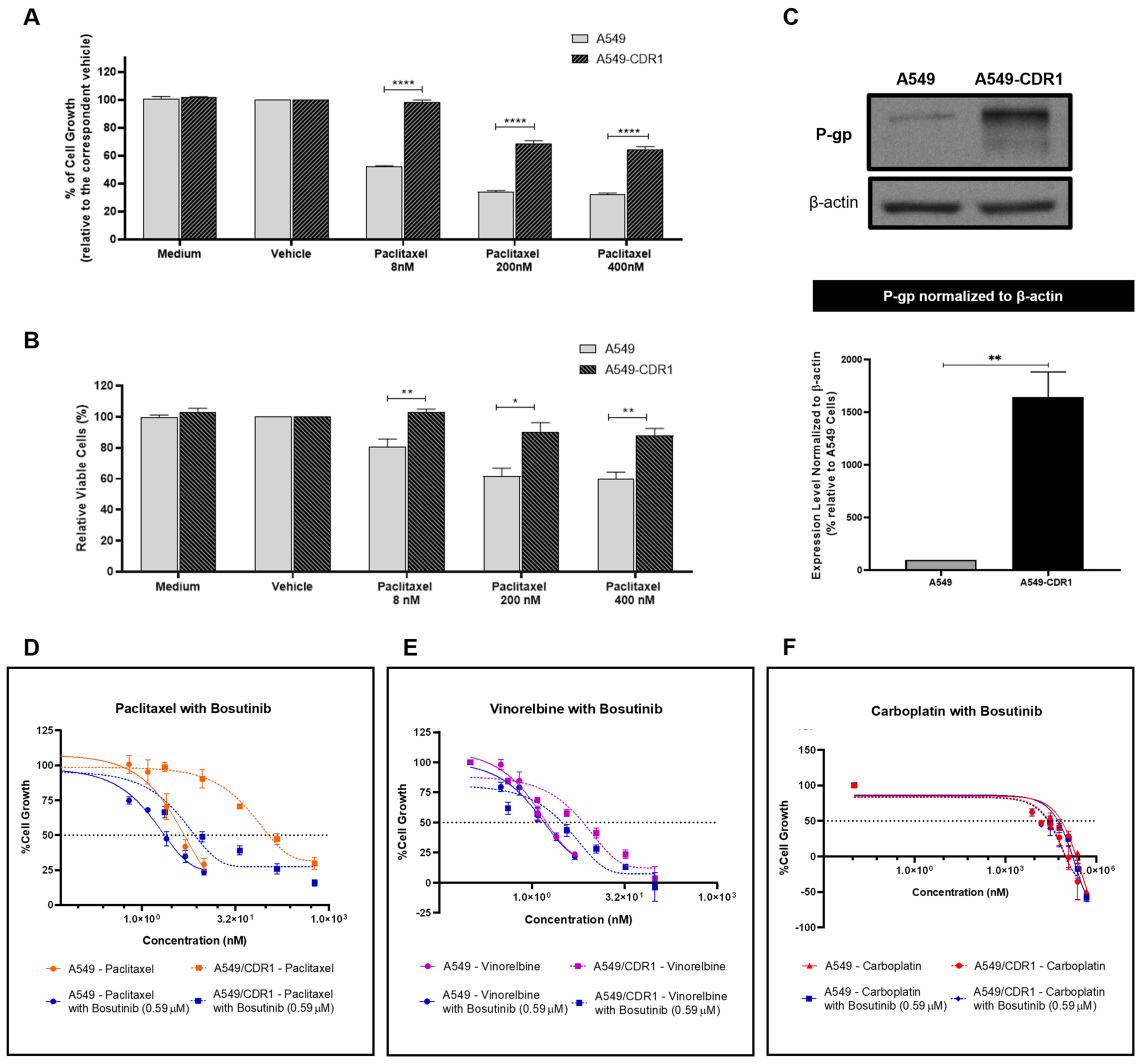
Confirmation of the MDR phenotype in the A549-CDR1 cell line and evaluation of the synergistic effect of bosutinib combined with conventional chemotherapeutic drugs in sensitive A549 and MDR A549-CDR1 cells. (A) Effect of different paclitaxel concentrations (8, 200, and 400 nM) on the % of cell growth of A549 and A549-CDR1 cells, assessed by the SRB assay. Vehicle at the highest concentration tested was used as control. Results are expressed as mean ± SEM of at least three independent experiments. Statistical significance was assessed using an unpaired Student’s *t*-test. ^****^P < 0.0001; (B) Effect of different paclitaxel concentrations (8, 200, and 400 nM) on cell viability (% of viable cells) of A549 and A549-CDR1 cells, assessed by trypan blue exclusion assay. Vehicle at the highest concentration tested was used as control. Results are expressed as mean ± SEM of at least three independent experiments. Statistical significance was assessed using an unpaired Student’s *t*-test. ^*^P < 0.05; ^**^P < 0.01; (C) P-gp expression in A549 and A549-CDR1 cells, assessed by WB. Protein levels were normalized to β-actin. Results are expressed as mean ± SEM of at least three independent experiments. ^**^P < 0.01. Representative blot shown; (D-F) Effect of serially diluted chemotherapeutic drugs: - paclitaxel (D), vinorelbine (E), and carboplatin (F), - alone or in combination with 0.59 µM bosutinib, on the % of cell growth of A549 and A549-CDR1 cells, assessed by the SRB assay. Vehicle at the highest concentration tested was used as control. Results are expressed as mean ± SEM of at least three independent experiments. MDR: Multidrug resistance; SRB: sulforhodamine B; SEM: standard error of the mean; P-gp: P-glycoprotein; WB: Western blotting.

To demonstrate that the observed effects were not cell-line specific, we tested A549 and A549-CDR1 as a second pair of cell lines. To confirm the resistant phenotype of the A549-CDR1 cell line, the effects of different concentrations of paclitaxel (8, 200, and 400 nM) on cell growth [Figure 5A] and viability [Figure 5B] were assessed in both sensitive and resistant cells. The A549-CDR1 cells exhibited significantly greater resistance, maintaining higher growth and viability than A549 cells under all conditions tested. Additionally, P-gp expression, analyzed by WB, was significantly increased in A549-CDR1 cells compared to A549 cells [Figure 5C], confirming their MDR phenotype.

The effect of bosutinib in combination with paclitaxel, vinorelbine, or carboplatin was further evaluated in this pair of sensitive and MDR NSCLC cell lines. The GI_50_ concentration of bosutinib for each cell line was first determined [Table 2], and a fixed concentration of 0.59 µM (corresponding to half of the lowest GI_50_ concentration) was selected for the combination assays.

In both A549 and A549-CDR1 cells, the combination of paclitaxel with bosutinib [Figure 5D and Table 4] resulted in a lower GI_50_ compared to monotherapy. Vinorelbine combined with bosutinib [Figure 5E and Table 4] reduced the GI_50_ only in the MDR cells, while carboplatin combined with bosutinib [Figure 5F and Table 4] lowered the GI_50_ in both cell lines. Notably, only the combinations containing paclitaxel or vinorelbine achieved statistical significance (*P* < 0.05) in the MDR cell line and demonstrated synergism (CI < 1, Table 4), validating our previous findings. In contrast, carboplatin combined with bosutinib showed no synergistic effect in either cell line (CI > 1, Table 4), which can be explained by the fact that carboplatin is not a P-gp substrate. A detailed statistical analysis of the effect of each concentration used in all drug combinations, compared to single-drug treatments and vehicle control, is provided in Supplementary Figure 2.

**Table 4:** GI_50_ concentrations (nM) of paclitaxel, vinorelbine, or carboplatin, alone or in combination with 0.59 µM bosutinib, in A549 and A549-CDR1 cell lines

	**A549**		***P*-value**	**A549-CDR1**		***P*-value**
**GI_50_ Concentration (nM)**	**Mean ± SEM**	**CI**		**Mean ± SEM**	**CI**	
Paclitaxel	2.95 ± 0.79			145.93 ± 26.23		
Paclitaxel with Bosutinib (0.59 µM)	2.53 ± 0.51	1.9	ns	9.87 ± 1.49	0.42	**
Vinorelbine	1.38 ± 0.21			10.93 ± 2.78		
Vinorelbine with Bosutinib (0.59 µM)	1.45 ± 0.22	1.61	ns	2.17 ± 0.64	0.64	*
Carboplatin	5.21 × 10^4^ ± 1.67 × 10^4^			4.30 × 10^4^ ± 2.69 × 10^4^		
Carboplatin with Bosutinib (0.59 µM)	3.86 × 10^4^ ± 1.45 × 10^4^	1.67	ns	3.03 × 10^4^ ± 1.77 × 10^4^	1.29	ns

CI and statistical significance of the combination *vs.* single-drug treatment were determined. Statistical significance was assessed using an unpaired Student’s *t*-test. ^*^P < 0.05; ^**^P < 0.01; ns: not significant. Results are expressed as mean ± SEM from at least three independent experiments. SEM: Standard error of the mean; CI: combination index.

### Bosutinib decreased p-caveolin-1 expression and modulated P-gp activity in the MDR NCI-H460/R cells

**Figure 6:**
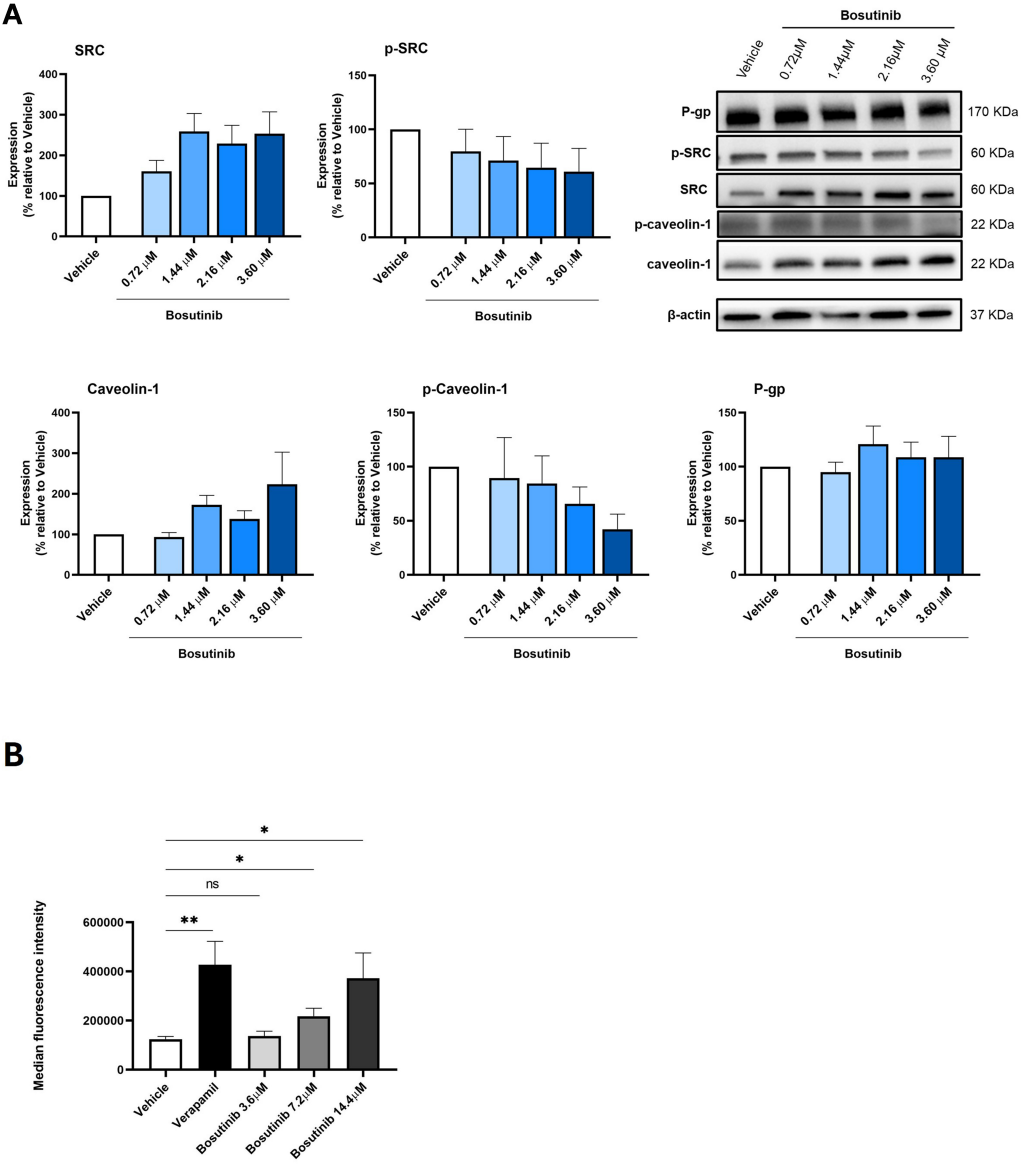
Effect of bosutinib on SRC, p-SRC, caveolin-1, p-caveolin-1, and P-gp in NCI-H460/R cells. (A) Protein expression levels were assessed by WB. Vehicle (DMSO at the highest concentration) was used as control. Results are expressed as the expression level of each protein normalized to β-actin, SRC (in the case of p-SRC) or caveolin-1 (in the case of p-caveolin-1). Data represent mean ± SEM of at least three independent experiments. Representative blots are shown; (B) Effect of different bosutinib concentrations on rhodamine-123 accumulation in NCI-H460/R cells, assessed by flow cytometry. Verapamil was used as a positive control. Vehicle (DMSO at the highest concentration) was included to exclude solvent toxicity. Results are represented as mean fluorescence intensity and are shown as mean ± SEM of at least three independent experiments. Statistical significance was determined using an unpaired Student’s *t*-test. ^*^P < 0.05; ^**^P < 0.01; ns: not significant. p-SRC: Phosphorylated form of SRC; P-gp: P-glycoprotein; WB: Western blotting; DMSO: dimethyl sulfoxide; SEM: standard error of the mean.

The effect of 48 h treatment with different concentrations of bosutinib on the expression levels of p-SRC, SRC, p-caveolin-1, caveolin-1, and P-gp in NCI-H460/R cells was assessed by WB [Figure 6A].

Following bosutinib treatment, SRC expression increased while p-SRC (the active form) decreased in MDR cells, as expected, since bosutinib is known to inhibit SRC phosphorylation^[35]^.

Similarly, bosutinib treatment induced a slight decrease in p-caveolin-1 and a slight increase in total caveolin-1, although these changes were not statistically significant [Figure 6A].

In contrast, bosutinib did not alter P-gp expression levels in NCI-H460/R cells [Figure 6A].

The effect of bosutinib on P-gp activity was further evaluated using the rhodamine-123 efflux assay, with verapamil as a positive control. Results indicated that increasing concentrations of bosutinib enhanced rhodamine-123 accumulation in the cells. Notably, treatment with bosutinib at 7.2 and 14.4 μM significantly decreased P-gp activity compared with vehicle-treated cells [Figure 6B].

### Bosutinib decreased the viability of NSCLC MDR 3D spheroids

**Figure 7:**
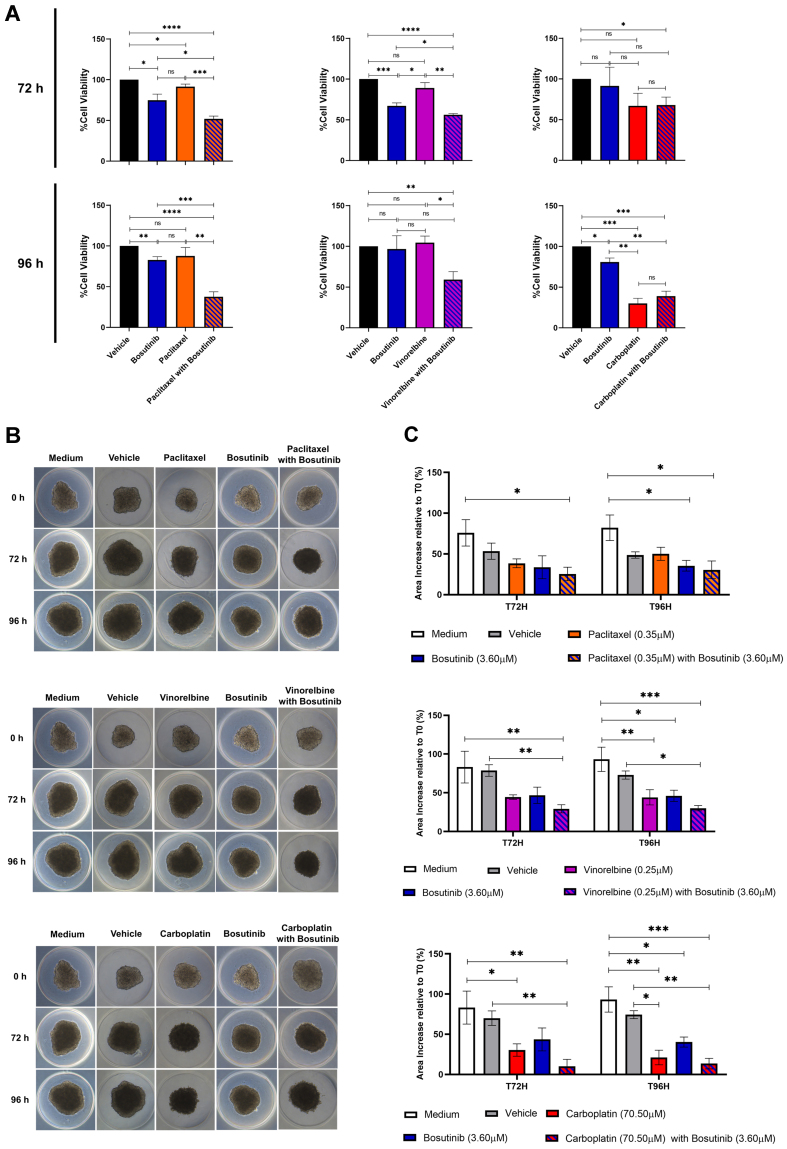
Effect of bosutinib in combination with paclitaxel, vinorelbine, or carboplatin on 3D spheroids from the MDR NSCLC NCI-H460/R cell line. (A) Spheroid viability after 72 and 96 h of drug treatment, assessed using the CellTiter-Glo® 3D Cell Viability Assay; (B) Representative spheroid morphology at the start of treatment (T0) and after 72 h (T72) and 96 h (T96) of drug treatment; (C) Quantification of spheroid area alterations after 72 and 96 h of drug treatment. Results are expressed as mean ± SEM of at least three independent experiments. Statistical significance was determined using an unpaired Student’s *t*-test. ^*^P < 0.05; ^**^P < 0.01; ^***^P < 0.001; ^****^P < 0.0001; ns: non-significant. MDR: Multidrug resistance; NSCLC: non-small cell lung cancer; SEM: standard error of the mean.

Finally, the effects of combining paclitaxel, vinorelbine, or carboplatin with bosutinib were evaluated in 3D spheroid models of the NCI-H460/R cell line after 72 and 96 h of treatment, using the CellTiter-Glo® 3D Cell Viability Assay [Figure 7A]. The combination of paclitaxel with bosutinib significantly reduced the viability of MDR spheroids compared to each drug alone at both timepoints. Similarly, the combination of vinorelbine with bosutinib was significantly more effective at decreasing spheroid viability than either drug alone, but only after 96 h of treatment. In contrast, the combination of carboplatin with bosutinib did not reduce spheroid viability more than carboplatin alone, consistent with the results obtained in 2D cell culture.

Interestingly, analysis of spheroid area [Figure 7B and C] showed that all drug combinations slowed spheroid growth compared to single-drug treatments and controls (vehicle and culture medium). This indicates that the drug combinations were more effective than individual drugs at limiting spheroid growth.

## DISCUSSION

Chemotherapy remains essential for the treatment of many NSCLC patients due to its cost-effectiveness and broad applicability. However, the development of MDR, often driven by overexpression of ABC transporters such as P-gp, continues to be a major obstacle in NSCLC therapy^[36]^. Therefore, identifying molecular targets to overcome MDR and developing more effective treatment strategies, including drug combinations, is crucial^[5]^. Tumor-derived EVs play a key role in intercellular communication by transporting molecules that influence recipient cells and contribute to oncogenic processes and chemoresistance^[19,37,38]^. Analyzing the protein content of MDR cells and their sensitive counterparts, alongside the protein cargo of EVs shed by MDR cells (here termed MDR-EVs) compared with EVs from sensitive parental cells (here termed sensitive EVs), may help identify proteins with differential expression. We hypothesized that the identification proteins - which are not shed into MDR-EVs but are rather selectively retained by MDR cells (differently from what happens in sensitive cells) - may be critical for establishing and/or maintaining the MDR phenotype. Such proteins could therefore serve as potential molecular targets to overcome MDR.

This study revealed significant differences in protein content between MDR and sensitive cells, as well as their respective EVs. Notably, the active phosphorylated form of SRC (p-SRC) was high in MDR cells but not in their EVs, indicating selective retention of this protein by resistant cells. Therefore, we propose that p-SRC retention contributes to the MDR phenotype. SRC is a well-studied oncoprotein that regulates key hallmarks of oncogenesis and chemoresistance^[39,40]^. Its overexpression and overactivation have been observed in numerous cancers and are associated with increased malignancy^[41]^. In our proteomic analysis, we also observed increased levels of CO-43 in MDR-EVs, despite similar expression in both sensitive and MDR cells. CO-43 is known to inhibit SRC activation^[33]^. These findings suggest that MDR cells preferentially secrete CO-43 via EVs, supporting the hypothesis of selective packaging of CO-43 into EVs as a way to prevent SRC phosphorylation. Importantly, SRC has been previously identified as a key mediator of chemoresistance^[42,43]^, highlighting this protein as a potential target to overcome drug resistance.

To investigate potential therapeutic strategies to overcome MDR, we explored the effect of two SRC inhibitors, bosutinib and ecF506, on sensitizing NSCLC MDR cells to chemotherapeutic drugs, namely paclitaxel, vinorelbine, and carboplatin. Bosutinib is an ATP-competitive SRC inhibitor approved by the Food and Drug Administration (FDA) for first-line treatment of newly diagnosed chronic myeloid leukemia^[34,44]^. EcF506 is a novel SRC inhibitor, not yet approved for clinical use, reported to block SRC in its inactive conformation, thereby inhibiting its phosphorylation^[45]^. Our results demonstrated that both inhibitors increased drug sensitivity in the pair of sensitive and counterpart MDR cells, NCI-H460 and NCI-H460/R, respectively. Notably, the synergistic effect of each inhibitor combined with the P-gp substrates paclitaxel or vinorelbine was stronger in MDR cells than in sensitive cells, with no effect observed for carboplatin, an ABCC2 substrate^[46]^. These findings were confirmed in another pair of NSCLC cells (A549/A549-CDR1). Furthermore, combinations including bosutinib (with paclitaxel, vinorelbine, or carboplatin) reduced the viability and growth of NCI-H460/R spheroids, supporting their potential to sensitize MDR NSCLC cells.

In fact, other studies demonstrate that SRC kinase plays a crucial role in MDR across different cancers by regulating drug efflux, survival signaling, and cellular adaptation mechanisms^[42,43,47]^. In lung cancer, the SRC inhibitor sunitinib reversed MDR in a cisplatin-resistant cell line, A549/DDP, by downregulating several efflux pumps, including P-gp, which led to increased intracellular drug accumulation and induced apoptosis^[47]^. Importantly, Yang *et al.* previously described a SRC-dependent modulation of P-gp in breast cancer^[43]^. In their study, SRC kinase indirectly interacts with P-gp through its binding to Rack1 and Anxa2. SRC phosphorylates Anxa2, which in turn enhances P-gp activity, leading to increased drug efflux and, consequently, MDR. This signaling cascade also drives cell proliferation and invasion, further promoting MDR mechanisms^[43]^. To our knowledge, interactions between SRC and efflux pumps other than P-gp have not yet been confirmed, which may explain why the synergistic effect of SRC inhibitors was stronger in cell lines overexpressing P-gp and treated with drugs that are its substrates. Taken together, these results suggest that the observed chemosensitizing effect occurs in a P-gp-dependent manner.

Fan *et al.* found that Rack1 and SRC play a crucial role in drug resistance in breast cancer cells by regulating P-gp activity^[42]^. Their study revealed that Rack1 acts as a scaffold protein, facilitating the binding of SRC to P-gp. This interaction allows SRC to phosphorylate Caveolin-1, which normally inhibits P-gp function. When Caveolin-1 is phosphorylated, its binding to P-gp is reduced, resulting in increased P-gp drug efflux activity and decreased therapeutic efficacy. Importantly, knocking down Rack1 or inhibiting SRC reduced P-gp activity, leading to greater intracellular drug retention and increased sensitivity to chemotherapy. These findings suggest that targeting Rack1 or interfering with the SRC–Caveolin-1–P-gp signaling pathway could be a potential strategy for overcoming drug resistance in breast cancer^[42]^. Based on these findings, we tested the effect of bosutinib on the expression levels of Caveolin-1 and p-Caveolin-1. Our data showed that NCI-H460/R cells treated with bosutinib exhibited decreased levels of phosphorylated Caveolin-1 (p-Caveolin-1), while total Caveolin-1 levels slightly increased. These results suggest that, similar to breast cancer^[42]^, inhibition of p-SRC by bosutinib may reduce p-Caveolin-1 levels, and, consequently, P-gp activity. Indeed, P-gp expression levels remained unchanged after bosutinib treatment, but the rhodamine-123 assay indicated a concentration-dependent decrease in P-gp activity. Thus, bosutinib reduced the transport activity of P-gp, consistent with published observations in breast cancer^[42]^.

Importantly, bosutinib combined with chemotherapeutic agents (paclitaxel, vinorelbine, and carboplatin) did not significantly increase cytotoxicity in non-tumorigenic cells, whereas combinations with ecF506 caused considerable toxicity. Although previous studies have shown that ecF506 effectively reduces the proliferation of breast cancer cells at sub-nanomolar concentrations^[48]^, to our knowledge its potential cytotoxicity in non-tumorigenic cell lines has not been explored. The clinical feasibility of bosutinib in NSCLC also warrants consideration. Although bosutinib reaches measurable plasma concentrations after oral administration and its pharmacokinetic profile is well established, it remains uncertain whether clinically attainable concentrations are sufficient to reproduce the chemosensitizing effects observed *in vitro*, particularly given its high protein binding and limited free-drug fraction. Safety aspects and potential drug-drug interactions may also influence its suitability when combined with standard chemotherapeutic regimens^[49]^. These factors highlight the need for careful evaluation of the clinical translational relevance of bosutinib in NSCLC.

Despite the novel insights provided by this study, several limitations should be acknowledged. First, the experiments were conducted using a limited number of established cancer cell lines. While these models are useful for initial investigations, they do not fully capture the extensive biological heterogeneity present in tumors from different patients. The genetic and phenotypic diversity among cancer patients is vast, and relying on a small panel of cell lines may limit the generalizability of our results. Therefore, including a wider variety of cell lines that represent different molecular subtypes would help broaden the applicability of bosutinib as a chemosensitizer. However, expanding the number of cell line panels is particularly challenging, as it requires generating matched resistant counterparts for each parental (sensitive) cell line. Establishing chemoresistant cell models is a time-consuming process that often takes several months, involving repeated drug-exposure cycles, careful monitoring of cell viability, and stepwise dose escalation. Each newly generated resistant line must also be thoroughly characterized to confirm the stability and specificity of the resistance phenotype. These procedures demand substantial technical effort, time, and resources, which significantly limits the feasibility of generating large panels of paired chemosensitive/chemoresistant cell lines. Second, although proteomic analyses provided important insights into the molecular pathways affected by bosutinib treatment, detailed mechanistic studies are still lacking. Our data identified several candidate proteins and pathways potentially involved in drug response and resistance, but the functional roles of these targets require further validation. Future research should focus on targeted experiments, such as gene knockdown or overexpression studies, to clarify the mechanisms through which bosutinib modulates chemosensitivity. Lastly, our *in vitro* data support the role of bosutinib as a chemosensitizer; however, the absence of *in vivo* validation prevents definitive conclusions about its therapeutic potential. Evaluating the combination of bosutinib with chemotherapeutic agents in resistant tumor xenograft models would significantly strengthen the translational relevance of our findings.

In conclusion, to the best of our knowledge, this is the first study suggesting that the selective retention of p-SRC by MDR cells, together with the reduced release of p-SRC in MDR-EVs, may contribute to chemoresistance in MDR NSCLC cells. Our work also identifies SRC as a potential molecular target to overcome MDR and supports the use of bosutinib - an FDA-approved SRC inhibitor - in combination with P-gp substrate chemotherapeutic drugs to sensitize MDR NSCLC cells. This proof-of-concept study provides a foundation for further preclinical investigations using advanced biological models, such as xenograft mouse models, and, if results are confirmed, for the development of a robust phase I/IIa clinical trial. Future work will also include mechanistic studies to elucidate the effects of bosutinib on the SRC–P-gp axis.

## DECLARATIONS

### Availability of data and materials

Raw data and some materials are available from the corresponding authors upon reasonable request.

### Copyright

© The Author(s) 2026.
