# Supplementary material for: Extracellular vesicles shed by multidrug resistant cells contribute to the identification of SRC inhibitors as chemosensitizers in non-small cell lung cancer
Source: Cancer Drug Resist. 2026 Feb 10;9:4. doi: 10.20517/cdr.2025.175 (PMC13103266; doi:10.20517/cdr.2025.175)
Supplement: Supplementary file 1 [file cdr-9-4-SupplementaryMaterials.pdf]

Supplementary Materials

Extracellular vesicles shed by multidrug resistant cells contribute to the identification of SRC inhibitors as chemosensitizers in non-small cell lung cancer

Bárbara Polónia[1,2,3,4,5], Cristina P. R. Xavier[1,2,6,7], Sara Peixoto da Silva[1,2,3], Chiara Riganti[4,5], M. Helena Vasconcelos[1,2,3]

[1]i3S - Instituto de Investigação e Inovação em Saúde, Universidade do Porto, Porto 4200-135, Portugal.
[2]Cancer Drug Resistance Group, IPATIMUP - Institute of Molecular Pathology and Immunology, University of Porto, Porto 4200-135, Portugal.
[3]Department of Biological Sciences, FFUP - Faculty of Pharmacy of the University of Porto, Porto 4050-313, Portugal.
[4]Department of Oncology, University of Torino, Torino 10126, Italy.
[5]Molecular Biotechnology Center "G. Tarone", University of Torino, Torino 10126, Italy.
[6]Associate Laboratory i4HB - Institute for Health and Bioeconomy, University Institute of Health Sciences - CESPU, Gandra 4585-116, Portugal.
[7]UCIBIO - Applied Molecular Biosciences Unit, Toxicologic Pathology Research Laboratory, University Institute of Health Sciences (1H-TOXRUN, IUCS-CESPU), Gandra 4585-116, Portugal.

Correspondence to: Prof. Cristina P. R. Xavier, Prof. M. Helena Vasconcelos, i3S - Instituto de Investigação e Inovação em Saúde, Universidade do Porto, Porto 4200-135, Portugal. E-mail: cristina.xavier@iucs.cespu.pt; hvasconcelos@ipatimup.pt; Prof. Chiara Riganti, Department of Oncology, University of Torino, Torino 10126, Italy. E-mail: chiara.riganti@unito.it

Supplementary Text 1: Additional parameters and filters used in the Proteome Discoverer 2.5.0.400 software for the proteomic analysis: i) all proteins included were identified in the *Homo sapiens* database; ii) all proteins exclusively identified in the *Bos taurus* database were excluded, as well as common contaminants; iii) the results were filtered considering "unique peptides" and "razor peptides" greater or equal to 2; iv) proteins were considered to be increased in the EVs derived from the NCI-H460/R cell line when the "abundance ratio" was greater than or equal to 2 in ratio "NCI-H460/R / NCI-H460", the "abundance ratio adjusted p-value" was less than or equal to 0.05 in ratio "NCI-H460/R / NCI-H460" and the "abundance (normalized)" was greater than or equal to 1 in all analysed samples of NCI-H460/R-EVs); v) proteins were considered to be decreased in the EVs derived from the NCI-H460/R cell line when the "abundance ratio" was less than or equal to 0.5 in ratio "NCI-H460/R / NCI-H460", the "abundance ratio adjusted p-value" was less than or equal to 0.05 in ratio "NCI-H460/R / NCI-H460" and the "abundance (normalized)" was greater than or equal to 1 in all analysed samples of NCI-H460-EVs.

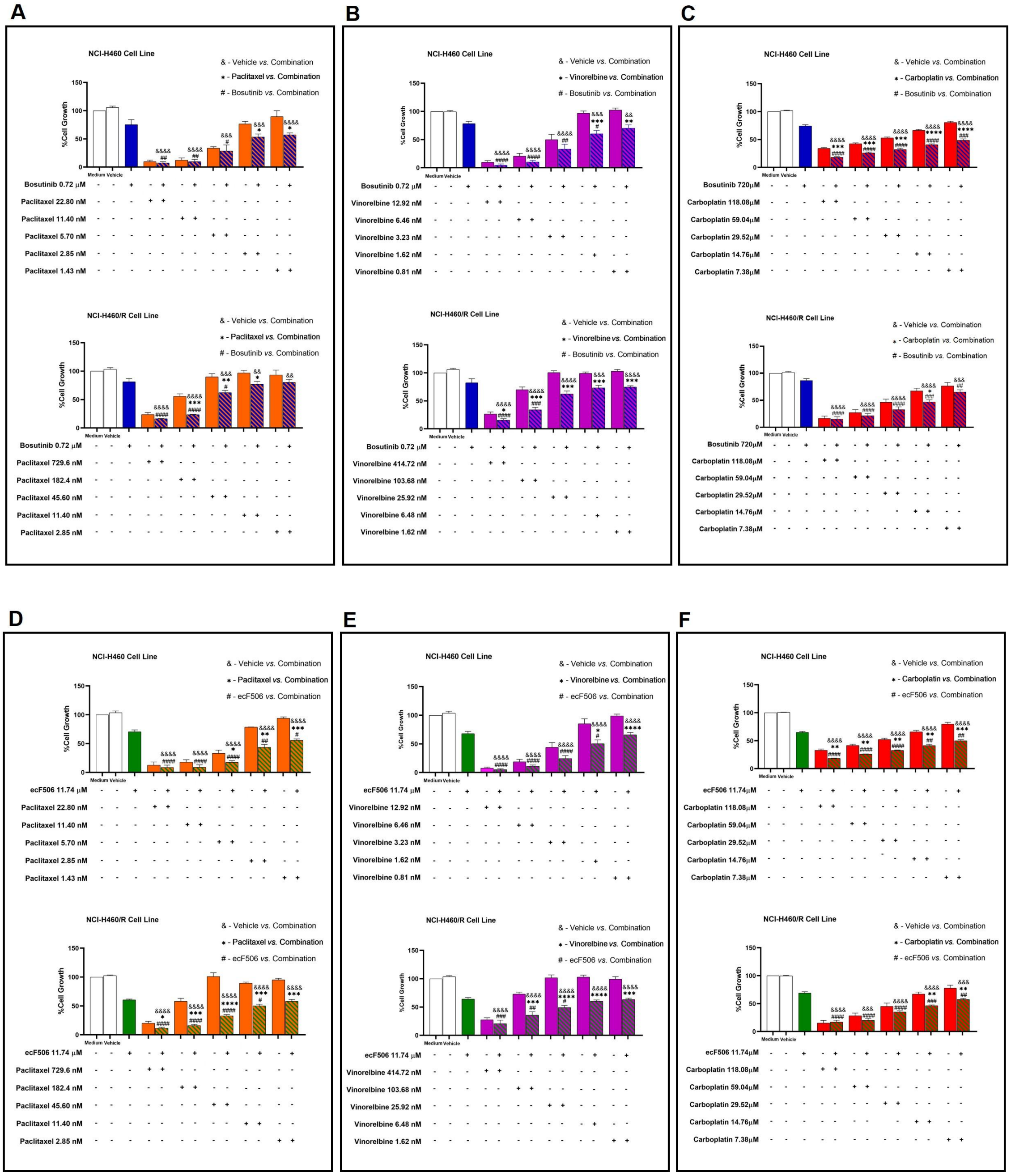

Supplementary Figure 1. Detailed statistical analysis performed for each cell line – NCI-H460 and NCI-H460/R - regarding the combination of five serial dilutions of (A) paclitaxel with 0.72 μM of bosutinib; (B) vinorelbine with 0.72 μM of bosutinib; (C) carboplatin with 0.72 μM of bosutinib; (D) paclitaxel with 11.74 μM of ecF506; (E) vinorelbine with 11.74 μM of ecF506; (F) carboplatin with 11.74 μM of ecF506. Results are represented as % of cell growth and are the mean ± SEM of at least three independent experiments. *, # or & $p < 0.05$; . **, ## or && < 0.01; ***, ### or &&& $p < 0.001$; ****, #### or &&&& $p < 0.0001$.

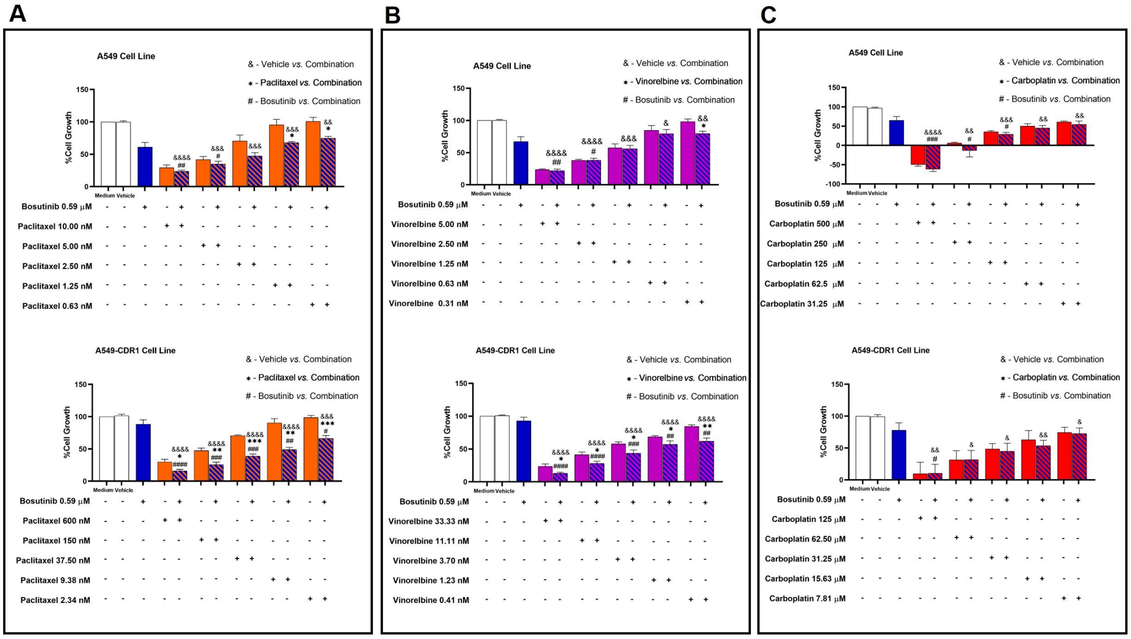

Supplementary Figure 2. Detailed statistical analysis performed for each cell line – A549 and A549-CDR1 – regarding the **c**ombination of of five serial dilutions of (A) paclitaxel with 0.59 µM of bosutinib; (B) vinorelbine with 0.59 µM of bosutinib; (C) carboplatin with 0.59 µM of bosutinib. Results are represented as % of cell growth and are the mean ± SEM of at least three independent experiments. *, # or & $p < 0.05$; . **, ## or && < 0.01; ***, ### or &&& $p < 0.001$; ****, #### or &&&& $p < 0.0001$.
